# Zeolites synthesis from phyllosilicates and their performance for CO_2_ adsorption

**DOI:** 10.1007/s11356-024-33685-0

**Published:** 2024-05-21

**Authors:** Salima Essih, Enrique Vilarrasa-García, Diana Cristina Silva Azevedo, Daniel Ballesteros-Plata, Isabel Barroso-Martín, Antonia Infantes-Molina, Enrique Rodríguez-Castellón, Francisco Franco, Juan Antonio Cecilia

**Affiliations:** 1 Department of Inorganic Chemistry, Crystallography, and Mineralogy, Faculty of Sciences, University of Málaga, Campus de Teatinos, 29071 Málaga, Spain; 2 GPSA - Grupo de Pesquisa Em Separações Por Adsorção, Departamento de Engenharia Química, Universidade Federal Do Ceará, Campus Do Pici, Fortaleza, 60455-760 Brazil

**Keywords:** Phyllosilicates, Kaolinite, Montmorillonite, 13X zeolite, 4A zeolite, CO_2_ adsorption

## Abstract

Five phyllosilicates (kaolinite, montmorillonite, saponite, sepiolite and palygorskite) have been selected as starting materials for the synthesis of zeolites. Among them, kaolinite and montmorillonite display the lowest Si/Al molar ratio leading to aluminosilicates with high crystallinity. Thus, the hydrothermal treatment under basic conditions forms 4A zeolite when kaolinite is used as starting material while 13X zeolite is obtained when montmorillonite is used as starting material. The microporosity and CO_2_-adsorption capacity of the prepared zeolites are directly related to its crystallinity. Thus, in order to improve it, raw phyllosilicates were subjected to a microwave-assisted treatment to remove undesired Mg or Fe-species, which have a negative effect in the assembling of the zeolites by hydrothermal basic conditions in a second step. The highest adsorption value was 3.85 mmol/g at 25 °C and 760 mm of Hg for Mont-A-B sample after the consecutive treatments.

## Introduction

The increase in anthropogenic CO_2_ emissions due to the ever-increasing population growth and energy demands, together with the massive consumption of fossil fuels, has aggravated global warming, causing severe effects in the world such as the melting of the polar ice shield and the rise in sea levels. Moreover, in some regions, extreme weather events and rainfall are becoming more common, while others are experiencing more extreme heat waves and droughts. Predictions are not very positive, since these impacts will intensify in the next decades if the CO_2_ content in the atmosphere continues to increase (Kessel 2000).

Taking into account the serious effects of greenhouse gases, governments are meeting to establish more restrictive legislations in order to diminish the CO_2_-anthropogenic emissions. The main challenge is the quest of alternative energy sources, away from traditional fossil fuels, to achieve net zero emissions. However, renewable energy sources are still developing technologies that cannot supply the world’s great energy demands. On the other hand, the design of more efficient processes in which CO_2_ emissions can be minimized is another challenge the scientific community is facing, yet these processes have only been studied on a small scale and have not been applied on a large scale due to the high energy demands. A proposed alternative until more efficient processes or more sustainable energy sources are found is the capture, storage and subsequent valorization of CO_2_ (Abdulla et al. 2021). In the CO_2_ capture and storage (CCS) processes, the most expensive step is CO_2_ capture (50–90% of the total cost), depending on the source of CO_2_ emission (Pera-Titus 2014). The most consolidated technology to capture CO_2_ from gaseous effluents streams is chemical absorption using alkylamines (Rochelle 2009), obtaining brilliant results in some processes as flue gas. Despite the high potential for CO_2_ capture, the intrinsic toxicity and corrosivity of the amine-derived compounds, the high energy demands in the regeneration step and the discontinuity of the process are limitations to their use (Aresta and Dibenedetto 2003). In recent years, less corrosive and toxic technologies have been developed, among which the use of membranes, cryogenic distillation or the use of adsorbents can be highlighted thanks to the remarkable results in CO_2_ capture. However, these technologies also display limitations related to the costs of large-scale implementation. In addition, their efficiency is diminished for streams with low CO_2_ concentration (Cerón et al. 2018; Font-Palma et al. 2021). In recent years, inorganic or organic–inorganic solids have emerged as an alternative to capture CO_2_ through physical or chemical interactions (Pera-Titus 2014). In this sense, it has been reported that some inorganic compounds with medium and strong basicity such as alkaline-earth oxides, can chemically capture CO_2_ molecules, since these oxides are prone to suffer carbonation processes. Even though these inorganic compounds are inexpensive and reach high CO_2_ uptakes, these materials have a serious drawback related to the high temperature required for their reuse due to the strong chemical interaction of CO_2_ molecules with these oxides (Grasa and Abanades 2006; Wang et al. 2012).

On the other hand, porous materials have also been used in CO_2_ adsorption processes since CO_2_ molecules can be trapped in narrow pores due to their high quadrupole moment. In this sense, metal–organic frameworks (MOFs) (Yu et al. 2017), covalent-organic frameworks (COFs) (Zeng et al. 2016) and graphene-organic frameworks (GOFs) (Haque et al. 2017) have shown excellent CO_2_-uptake at laboratory scale, thanks to their narrow and modulable channels. However, the cost for pilot-scale is too high to be sustainable and competitive.

Porous silicas with different morphologies have been proposed for CO_2_ capture. However, CO_2_ uptakes with these materials are relatively low, therefore some methodologies like grafting and impregnation have been proposed to incorporate amine species and improve the adsorption capacity by increasing the amount of chemical adsorption sites (Cecilia et al. 2020; Chen et al. 2017; Vilarrasa-García et al. 2020; Yan et al. 2011). Porous activated carbons are other thoroughly studied adsorbents that exhibit high adsorption values thanks to their narrow pore size and high surface area. Nonetheless, the use of low cost starting materials is required to achieve sustainable and competitive processes (Abuelnoor et al. 2021; Chouikhi et al. 2021; Pevida et al. 2008; Serafin et al. 2017). Zeolites are hierarchical aluminosilicates with a narrow pore size that, like activated carbons, can also retain a large amount of CO_2_ (Boycheva et al. 2021; Murge et al. 2019; Zagho et al. 2021). For the synthesis of zeolites, a source of silicon and aluminum is required in a basic medium under hydrothermal conditions (Derbe et al. 2021; Koohsaryan and Anbia 2016). Considering that CO_2_ capture is the most expensive step in the CCS process, the use of low-cost silicon and aluminum sources is necessary to obtain inexpensive zeolites with high CO_2_-adsorption capacity (Khaleque et al. 2020). In this sense, several raw materials have been proposed such as blast furnace slag (Sugano et al. 2005), rice husk ash (Mohamed et al. 2015; Saceda et al. 2011), paper sludge ash (Mun and Ahn 2001), coal fly ash (Amoni et al. 2019), waste of iron mine tailings (Zhang and Li 2018), waste glass materials (Tsujiguchi et al. 2014) or minerals and rocks such as obsidian (Belviso 2016; Mamedova 2016), perlite (Filho et al. 2018; Wajima and Onishi 2019) or clay minerals (Abdullahi et al. 2017; Belviso et al. 2017; Johnson and Arshad 2014; Mackinnon et al. 2010; Youssef et al. 2008). In the present research, several phyllosilicates (kaolinite, montmorillonite, saponite, sepiolite and palygorskite) have been used as starting materials for the synthesis of zeolites through the alkaline fusion method (Belviso et al. 2017; Chen et al. 2014; Khalifa et al. 2000). To remove the potential impurities of the starting phyllosilicates, the samples were subjected to a microwave-assisted acid treatment, since this treatment causes a partial leaching of the phyllosilicates in short times (Cecilia et al. 2018a; Franco et al. 2016, 2020). Once the materials were synthesized by hydrothermal treatment under basic conditions, the obtained samples were tested in CO_2_ adsorption processes.

## Materials and methods

### Starting materials

Five clay minerals (kaolinite, montmorillonite, saponite, sepiolite and palygorskite) were selected as starting materials to the synthesis of zeolites. These materials were treated with a microwave-assisted acid treatment to modify their chemical composition due to a partial leaching of the octahedral sheet (Cecilia et al. 2018a; Franco et al. 2016, 2020).

The synthesis of potential zeolites from raw clay minerals and those modified by microwave-assisted acid treatment was carried out through hydrothermal treatment under basic conditions, following the methodology described in previous work (Cecilia et al. 2022). Briefly, 1 g of raw clay or a microwave-assisted acid-modified clay mineral was treated with a solution of 40 mL of NaOH (2 M) in a Teflon-lined stainless-steel autoclave for 48 h at 100 °C. The obtained material was centrifuged and washed several times to remove Na^+^ excess. Finally, the samples were dried at 80 °C overnight.

The samples were labeled with the initials Kao for kaolinite, Mont for montmorillonite, Sap for saponite, Sep for sepiolite and Pal for palygorskite. The nomenclature clay-A was used for the clay treated by microwave-assisted acid treatment, clay-B for the raw clay subjected to hydrothermal treatment under basic conditions and clay-A-B for those clays modified by microwave-assisted acid treatment, and then by hydrothermal treatment under basic conditions.

### Characterization of the materials

The crystalline structure of the clays and potential zeolites was determined by X-ray diffraction (XRD) using a PANalytical X’Pert PRO equipment and recorded in Bragg–Brentano reflection geometry (θ/2θ). This diffractometer is equipped with a Ge (111) monochromator to use monochromatic Cu Kα_1_ radiation (λ = 1.54059 Å) strictly.

The quantification of the main elements of the adsorbents was carried out in a MagiX X-ray fluorescence (XRF) spectrometer supplied by PANanalytical. A Varian 220-FS QU-106, atomic absorption (AA) spectrometer was used for the determination of Na-species. Loss of ignition (LOI) was determined at 950 °C.

The morphology of the particles was examined by scanning electron microscopy (SEM) using a JEOL SM-6490 LV combined with X-ray energy dispersive spectroscopy (EDX). The samples for SEM observation were previously gold sputtered to avoid charging of the surface.

The obtained materials were also characterized by attenuated total reflection (ATR) in a FT-IR Vertex70 spectrophometer (Bruker), showing a single reflection gold-gate diamond ATR system. For the acquisition of each spectrum, a standard resolution of 4 cm^−1^, 64 accumulations and a spectral range between 4000 and 500 cm^−1^ were used.

The textural properties of the microporous materials were determined from CO_2_ adsorption–desorption at 0 °C. CO_2_ was selected to analyze the textural properties instead of N_2_ since this gas is more appropriate to analyze the microporosity due to the hindered access to narrow micropores of N_2_-molecules. The micropore volume was calculated through the Dubinin-Radushkevich equation (Dubinin and Radushkevich, 1947). In all cases, the samples were previously outgassed at 200 °C and 10^−4^ mbar for 12 h.

The prepared materials were also characterized by solid-state NMR. For these samples, ^29^Si and ^27^Al MAS NMR spectra were analyzed in a Bruker AVIII HD 600 NMR equipment (field strength of 14.1 T) at 156.4 MHz with a 2.5 mm triple-resonance DVT probe that used zirconia rotors at the spinning rates of 15 kHz (^29^Si) and 20 kHz (^27^Al). ^29^Si MAS NMR analysis was carried out with proton decoupling (continuous wave sequence), applying a single pulse of π/2, an excitation pulse of 5 µs and a 60 s relaxation delay to obtain 10,800 scans. ^27^Al MAS NMR analysis were also carried out with proton decoupling (continuous wave sequence), applying a single pulse of π/12, an excitation pulse of 1 µs, and a 5-s relaxation delay to obtain 200 scans. The chemical shifts were referenced to as an external solution of tetramethylsilane for ^29^Si and to an external solution of 1 M of Al(NO_3_)_3_^27^Al, respectively.

### CO_2_ adsorption studies

To evaluate the CO_2_ adsorption capacity of the raw clays, the clays treated by microwave-assisted acid treatment and the clays treated under hydrothermal conditions in a basic medium, the samples were degassed at 150 °C for 12 h. Then, the analyses were carried out at 25 °C, between 0 and 760 mm of Hg using a Micromeritics 2420 apparatus.

In a next step, the isotherms were fitted to a Toth model.

$${q}_{i}=\frac{{q}_{mi}({b}_{i}{P}_{i})}{(1+({b}_{i}{P}_{i}{)}^{{t}_{i}}{)}^{{~}^{1}\!\left/ \!{~}_{{t}_{i}}\right.}}$$

where *q*_*m*_ is the maximum adsorbed concentration, *b* is the affinity parameter between adsorbent and adsorbate and *t* represents the surface heterogeneity of the adsorbent.

The accuracy of each fit was carried out for the Average Relative Error:

$$ARE\left(\%\right)=\frac{1}{{N}_{T}}{\sum }_{i=1}^{{N}_{T}}\frac{\left|{q}_{i,exp}-\left.{q}_{i.est}\right|\right.}{{q}_{i,exp}}*100$$

where *N*_*T*_ is the total number of the data points, and *q*_*i,est*_ and *q*_*i,exp*_ are the estimated and experimental amounts of CO_2_ adsorbed, respectively.

## Characterization of the samples

**Fig. 1:**
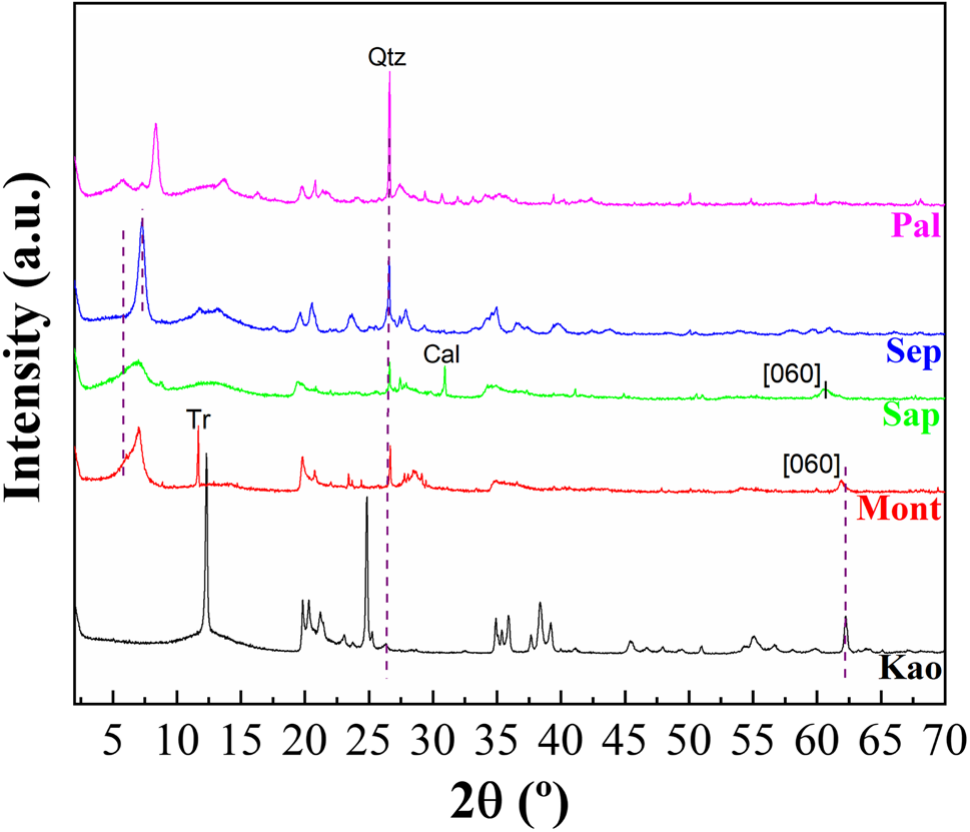
X-ray diffractograms of the starting raw clays: Kaolinite (Kao), Montmorillonite (Mont), Saponite (Sap), Sepiolite (Sep) and Palygorskite (Pal)

The analysis of crystallinity and the identification of crystalline phases in the raw clays were determined by XRD (Fig. 1). In the case of the Kao sample, the diffractogram shows higher crystallinity compared to the other samples, with two well-defined reflections located around 2θ of 12 and 25°, which are assigned to [001] and [002] reflections of kaolinite (Franco et al. 2014). Bragg equation applied to [001] reflection reveals that this sample displays a basal spacing of 7.2 nm. The presence of another set of diffraction peaks located at 34–36, 38–42, 45–50 and 54–63° also stands out, whose intensity depends on the origin of the kaolinite (Franco et al. 2014). On the other hand, the signal located at 2θ of 62.3° (1.49 nm) is ascribed to the [060] reflection and confirms the presence of a dioctahedral clay of the kaolin group. Finally, the presence of a small signal located at 2θ of 26.2° is also noted, which is assigned to small impurities of quartz.

The diffractogram of the Mont sample (Fig. 1) shows a material with less crystallinity than the Kao sample. The [001] reflection located at the lowest 2θ value shows two bands, which indicates a variability in the hydration of the interlayer space (12.6–15.0 nm). In addition, as observed in the Kao sample, a diffraction peak located at 62.3° (1.49 nm) stands out, which confirms the presence of a dioctahedral smectite such as montmorillonite (Cecilia et al. 2018a). Finally, the presence of the main diffraction peak of quartz (Qtz) and tremolite (Tr) as impurities is also observed.

For the Sap sample (Fig. 1), the intensity of the signals is lower, suggesting a sample with poorer crystallinity. A broad diffraction peak is also observed at lower 2θ values, so the presence of variable H_2_O-content is expected. The analysis of the [060] reflection located at 2θ of 60.9° (1.52 nm), which is typical of a trioctahedral smectite as saponite (Cecilia et al. 2018a). Regarding the impurities, small proportions of quartz and calcite (Cal) can be observed.

In the case of the Sep sample, the typical diffraction peaks of sepiolite are detected (Franco et al. 2014), although an impurity of quartz is also observed. Regarding the Pal sample, the diffractogram confirms the presence of palygorskite (Pardo-Canales et al. 2020) with the presence of small impurities of smectite, sepiolite and quartz.

In the synthesis of zeolites, a drawback is the presence of Mg- and Fe-species, since these species are not soluble in a basic medium, so they can negatively affect the assembly of silicate and aluminate to form zeolites. It is well known that trioctahedral clays have a high Mg-content. These Mg-species can be removed under acid treatment by leaching. In the same way, microwave-assisted acid treatment accelerates and increases the efficiency of partial leaching in trioctahedral clays (Cecilia et al. 2018a).

**Fig. 2:**
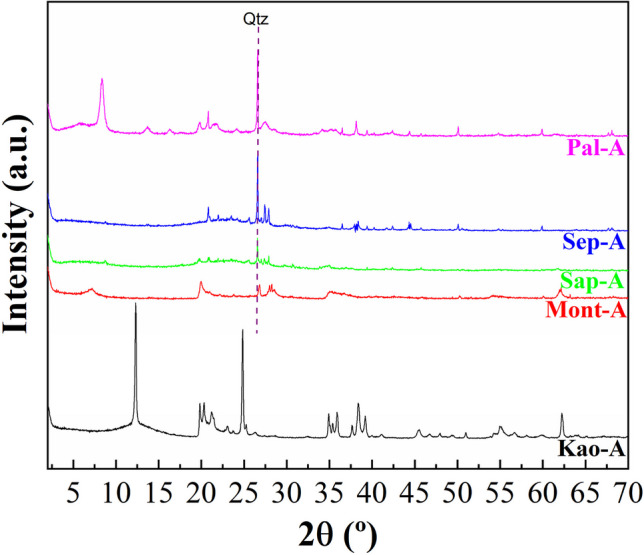
X-ray diffractograms of the phyllosilicates subjected to microwave-assisted acid treatment

**Fig. 3:**
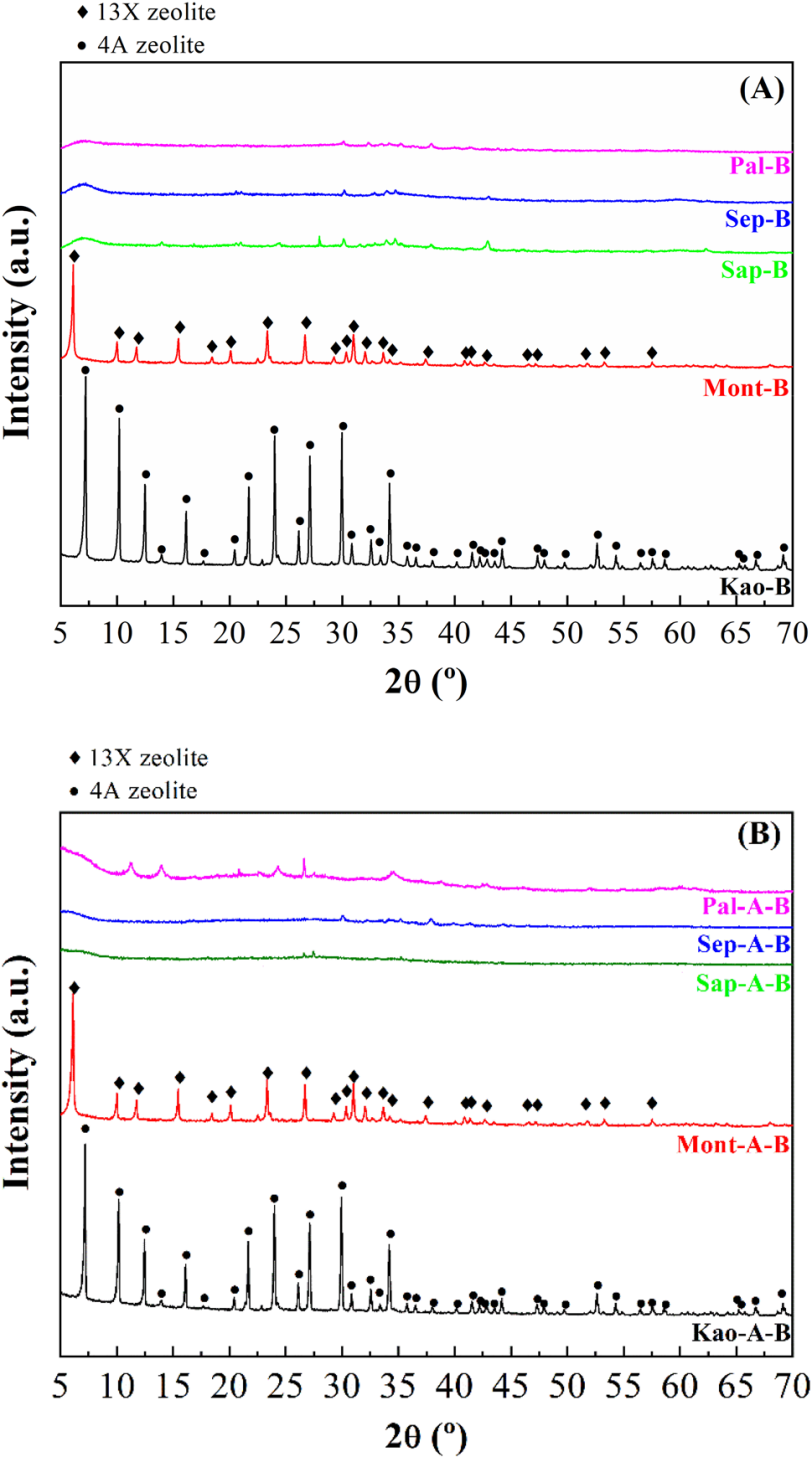
X-ray diffractograms of the aluminosilicates obtained after the hydrothermal treatment in basic medium of the raw phyllosilicates (**A**) and phyllosilicates subjected to microwave-assisted acid treatment and then a hydrothermal treatment in basic medium (**B**)

The possible modification of the crystallinity in the clays after microwave-assisted acid treatment for 16 min using 0.2 M HNO_3_ solutions was evaluated by XRD and registered diffractograms are displayed in Fig. 2. The study of the kaolinite samples after the acid treatment (Kao-A) shows that this sample is barely affected by microwave-assisted acid treatment, confirming that the trioctahedral TO structures are highly resistant to acid treatment. Similarly, the acid treatment of the Mont sample (Mont-A) hardly modifies the crystallinity of the sample since its TOT structure is maintained, although under the acid treatment some impurities such as tremolite are removed. Regarding the Sap sample treated with microwave-assisted acid treatment (Sap-A), the intensity of the diffraction peaks decreases notably. In this sense, the absence of the [001] reflection, which should appear at 2θ of 7°, suggests that the lamellar structure of saponite disappears. Similarly, acid treatment also removes the impurity of CaCO_3_. However, other impurities such as quartz seem to be more resistant to acid treatment. A similar trend was observed for Sep-A sample, where the [110] reflection also disappears after acid treatment, suggesting that the fibrous structure of the sepiolite is seriously damaged. In the case of palygorskite treated with microwave-assisted acid treatment (Pal-A), the [110] reflection is maintained, evidencing that the structure of palygorskite seems to be more resistant than that of sepiolite. In this sample, the impurities ascribed to quartz and smectite remain, so the smectite can be a dioctrahedral clay as montmorillonite due to its resistance to acid treatments. When the raw clays and the acid treatment-modified clays are subjected to a hydrothermal treatment in a basic medium, the diffractograms evidence that crystalline phases are only obtained from kaolinite or montmorillonite as starting materials (Fig. 3A and B). The use of saponite, sepiolite or palygorskite as starting materials to synthesize zeolites leads to amorphous materials even when the samples are subjected to microwave-assisted acid treatment. Regarding crystalline materials, two diffraction profiles can be observed. When the starting clay is kaolinite, the diffraction peaks are assigned to the formation of zeolite 4A (PDF N. 01–075-1151) while the use of montmorillonite as starting clay leads to the typical diffraction peaks of zeolite 13X (PDF N. 00–038-0237). In all cases, although a high crystallinity of the synthesized zeolites is observed, the presence of amorphous phases should not be ruled out.

To evaluate the modifications in the chemical composition of clays and potential zeolites to be used in CO_2_ capture processes, XRF studies were carried out (Table 1). The study of raw clay minerals by XRF is directly related to the results obtained by XRD. Thus, it can be observed how Kao is an aluminosilicate where the Mg-content is very low, which confirms a dioctahedral clay, as already observed in the [060] reflection. The absence of alkaline cations suggests the presence of a TO structure, while the low proportion of other elements confirms the absence of impurities. In the case of the Mont sample, it can be observed that the clay is Al-rich smectite, as suggested by XRD, although a small proportion of Mg-species is also noted. On the other hand, the presence of alkaline cations suggests the presence of a TOT structure where the cations are located in the interlayer space. Regarding the Sap sample, the Mg content is much higher than the Al content, leading to a trioctahedral clay. The presence of Ca-, K- and Na-species, which must be in the interlayer space, suggests that this material is a Mg-smectite. In the case of the fibrous clays (Sep and Pal), it can be observed how the Sep sample displays a higher Mg-content than Pal sample. In both samples, the percentage of Na-, K- or Ca-species is very low, discarding the presence of a high proportion of smectites or the inclusion of these cations in the microchannels of the fibrous structure.

**Table 1:** Chemical composition estimated by XRF of the raw phyllosilicates, phyllosilicates subjected to microwave-assisted acid treatment, phyllosilicates subjected to hydrothermal treatment in basic conditions and phyllosilicates subjected to microwave-assisted acid treatment and then hydrothermal treatment in basic conditions

Sample	SiO_2_	Al_2_O_3_	K_2_O	Fe_2_O_3_	TiO_2_	MgO	Na_2_O	P_2_O_5_	SO_3_	Cr_2_O_3_	CaO	MnO	Cl	LOI	Si/Al
Kao	52.60	32.29	0.60	0.53	0.30	0.19	0.08	0.03	0.03	0.03	0.02	-	-	13.23	1.38
Kao-A	48.91	34.82	-	0.26	2.02	-	-	0.02	-	0.02	-	-	-	13.86	1.19
Kao-B	28.66	22.80	0.02	0.24	1.21	-	28.85	-			0.02	-	-	18.20	1.06
Kao-A-B	32.50	23.95	0.04	0.79	1.16	0.03	21.88	-	0.01	-	0.03	-	-	19.55	1.15
Mont	58.18	15.84	0.40	5.47	0.33	2.69	4.31	0.04	1.62		1.91	0.01	0.33	8.71	3.17
Mont-A	62.44	16.14	0.31	4.59	0.31	2.63	0.70	0.01	0.05	-	0.48	0.01	-	12.26	3.27
Mont-B	38.10	9.16	0.27	3.31	0.18	1.47	28.91	0.02	0.84	-	1.26	0.02	0.22	16.24	3.53
Mont-A-B	32.78	6.79	0.17	3.67	0.15	1.40	37.54	-	0.02	-	0.43	-	-	17.05	4.09
Sap	49.08	6.25	1.37	2.94	0.41	19.70	3.92	0.06			2.66	0.06		13.51	6.66
Sap-A	75.11	4.28	1.29	2.60	0.51	2.12	0.37	0.02			0.13	0.04		13.79	14.89
Sap-B	37.08	3.35	1.04	2.92	0.32	10.14	29.54	0.05	0.02		1.70	0.05		1.35	9.35
Sap-A-B	39.72	1.94	0.68	2.66	0.23	1.09	34.69				0.08	0.03		18.88	17.44
Pal	57.64	6.55	0.28	5.21	0.42	8.66	0.11	1.34	0.02	0.07	5.05	0.02		14.55	7.46
Pal-A	68.17	5.91	0.26	4.05	0.46	6.70		0.08		0.05	0.25	0.01		14.01	9.76
Pal-B	40.38	6.88	0.18	4.05	0.27	5.29	24.47	0.82	0.03	0.05	3.77	0.02		13.79	4.95
Pal-A-B	44.40	7.95	0.08	6.54	0.61	9.54	15.89				0.34	0.02		14.55	4.72
Sep	58.98	2.30	0.79	2.32	0.12	21.51	0.19	0.02			0.67	0.06		13.00	21.67
Sep-A	77.74	2.06	1.05	0.26	0.15	1.94	0.42				0.13	0.02		16.21	31.96
Sep-B	39.82	2.09	0.64	0.57	0.11	13.66	25.69	0.02			0.58	0.04		16.78	16.23
Sep-A-B	46.94	1.41	0.68	0.35	0.08	0.82	31.31				0.09			18.32	28.23

Regarding possible modifications due to microwave-assisted acid treatment, it can be observed how the Al-rich phyllosilicates are highly resistant to acid treatment with microwave, as in the XRD (Figs. 1 and 2) where the Kao and Mont samples hardly suffer modifications after the acid treatment. However, trioctahedral clays, i.e. those phyllosilicates with higher Mg-content, suffer a clear loss of Mg-content when the samples are undergone to microwave-assisted acid treatment (Cecilia et al. 2018a; Franco et al. 2016). This leaching may cause a collapse in the lamellar structure in Sap sample and the fibrous structure in Sep sample, leading to amorphous materials for Sap-A and Sep-A, as observed by XRD (Figs. 1 and 2). In the case of those clays with Ca species, leaching of Ca species is also observed after acid treatment. The loss of Mg and Ca also affects the purification of clays since some crystalline phases such as calcite or tremolite are removed after acid treatment. On the other hand, it is also striking that the Fe-species are resistant to acid treatment.

After hydrothermal treatment in a basic medium to try the synthesis of the zeolites, the atomic concentration, determined by XRF, varies notably, increasing the Na-content significantly (Table 1). It is necessary to remember that the zeolitization process takes place through the following steps: (I) dissolution of the aluminosilicate into aluminate and silicate species; (II) condensation of the aluminate and silicate species forming oligomers; and (III) assembly and growth of oligomers to give rise to highly ordered three-dimensional structures.

In the case of the Kao and Kao-A samples, where the Si/Al molar ratio is the highest, the absence of impurities, mainly Mg-species, forms an aluminosilicate with a well-defined structure (zeolite 4A) (Fig. 3A). When Mont and Mont-A are used as starting materials to synthesize the zeolite, the assembly of the silicate and aluminate oligomers leads to another three-dimensional distribution forming a 13X zeolite (Fig. 3A), given the different Si/Al molar ratio for Kao and Mont.

Regarding the other clay minerals (Sap, Sep and Pal), the Si/Al molar ratio is higher than that observed for Kao and Mont, so the oligomerization and assembly must be different for these phyllosilicates. Moreover, the presence of a high Mg content, which is insoluble under basic conditions, also has an adverse effect on the assembly of the oligomers to synthesize the zeolites, obtaining in these cases an amorphous aluminosilicate (Fig. 3A). The microwave-assisted acid treatment aimed to minimize the Mg content but has an adverse effect on the synthesis of zeolites (Fig. 3B). Moreover, the Si/Al molar ratio is quite far from that of the zeolites synthesized from Kao and Mont, which are highly crystalline. Thus, the low Al content of Sap, Sep and Pal seems to have an adverse effect on the assembly of crystalline zeolites.

**Fig. 4:**
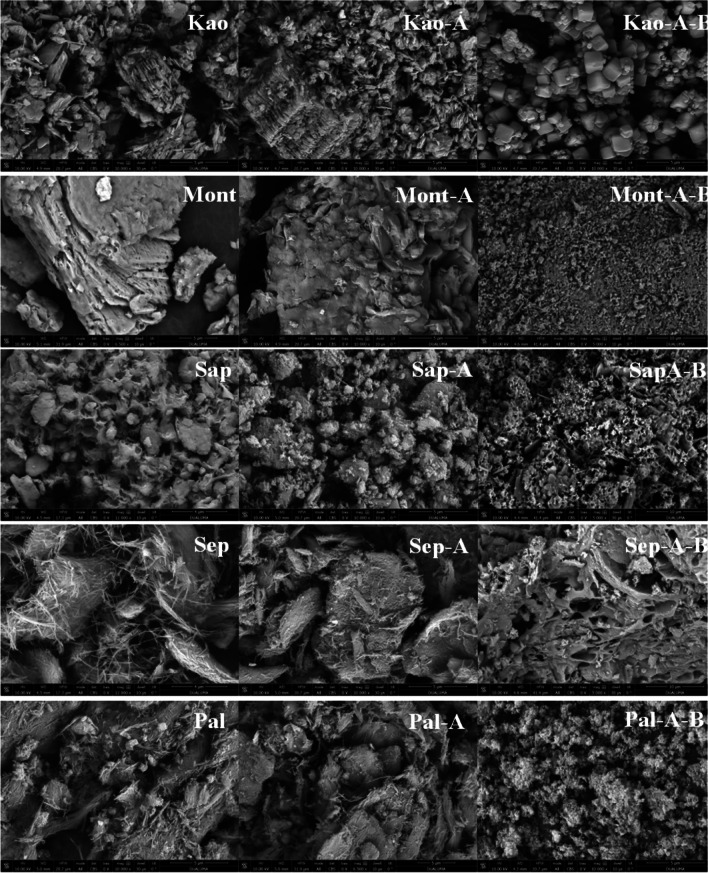
SEM images of raw phyllosilicates, phyllosilicates subjected to microwave-assisted acid treatment and, phyllosilicates subjected to microwave-assisted acid treatment and then hydrothermal treatment in basic medium. Scale: 5 µm

To analyze the morphology of raw clays, clays modified under microwave-assisted acid treatment, and amorphous aluminosilicates or zeolites synthesized by hydrothermal treatment under basic conditions, SEM images were performed (Fig. 4). The SEM image of the natural kaolinite confirms the existence of laminar habits, although it can be observed that these laminar structures present a house of cards structure as a consequence of the hydrothermal alterations to which the clay is subjected as well as the grinding treatment. After the microwave-assisted acid treatment, SEM image shows that the sample partially maintains a lamellar structure, evidencing a mixture of large stacks of kaolinite with lamellar structures smaller than those of the starting materials. The most striking change occurs when the zeolite is treated under hydrothermal and strongly basic conditions, since the lamellar structure disappears, giving rise to particles with cubic morphology. The structural changes agree with those observed by XRD where a highly crystalline zeolite (4A) is formed after hydrothermal treatment under basic conditions (Fig. 3B).

In the case of the Mont sample (Fig. 4), the image shows particles of variable size formed by stacked sheets, confirming the laminar structure suggested by XRD (Fig. 1). The acid treatment only causes slight modifications in the particles since it seems to affect the edges of the sheets in such a way that the acid treatment under these conditions hardly affects the montmorillonite structure, which agrees with the XRD data (Fig. 2). However, the basic treatment causes a strong modification in the morphology and size of the particles, obtaining small particles with a diameter of less than 1 µm, which must be highly ordered according to XRD (Fig. 3B), where zeolite 13X is observed.

For the Sap sample (Fig. 4), a low ordering material is observed, which seems to agree with the XRD data where the order is relatively low in comparison to other TOT smectites such as montmorillonite. The microwave-assisted acid treatment leads to a material with a different morphology from that of the starting material, confirming the efficiency of the acid treatment for the saponite and obtaining a material with poor crystallinity as detected by XRD (Fig. 2). Hydrothermal treatment under basic conditions also modifies the structure of the sample, leading to smaller particles with variable morphology.

In the case of the fibrous clays (Fig. 4), a different behavior can be observed after microwave assisted acid treatment since sepiolite is more vulnerable to acid treatment than palygorskite, as previously observed in literature (Cecilia et al 2018b). After hydrothermal treatment under basic conditions both materials also suffer drastic changes, obtaining small particles, which in the case of the sample from Pal are piled up, while in that from Sep large cavities are noticeable.

**Fig. 5:**
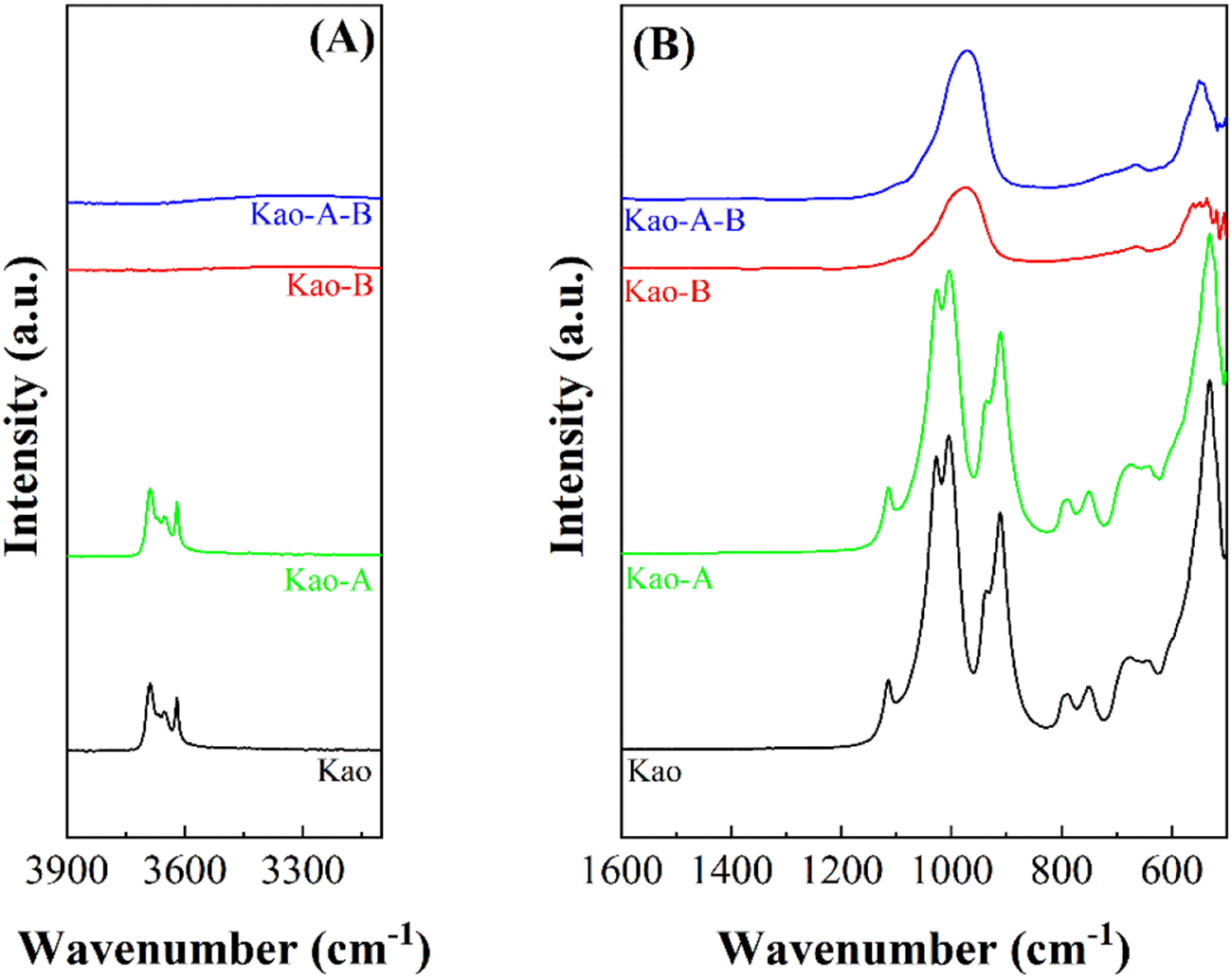
ATR spectra of kaolinite (Kao), kaolinite subjected to microwave-assisted acid treatment (Kao-A), kaolinite subjected to hydrothermal treatment in basic medium (Kao-b) and kaolinite subjected to microwave-assisted acid treatment and then hydrothermal treatment in basic medium (Kao-A-B)

The samples were also characterized by ATR. The hydroxyl region of the Kao sample shows four bands (Fig. 5A): one at 3688 cm^−1^, assigned to the in-phase symmetric stretching vibration; two weaker bands located at 3671 and 3652 cm^−1^ which are attributed to out-of-plane stretching vibration modes; and a last band located at 3620 cm^−1^, ascribed to the inner hydroxyl groups located between the tetrahedral and octahedral sheets (Madejova 2003). In the region between 1300 and 400 cm^−1^ (Fig. 5B), several well-defined and strong bands can be observed between 1150 and 1000 cm^−1^, which are assigned to Si–O stretching modes. The bands located between 970 and 850 cm^−1^ are assigned to Al_2_-OH bending modes of the dioctahedral clays (Madejova 2003) while the bands located at 795 and 745 cm^−1^ are assigned to Si–OH-Al bending modes (Madejova 2003). The weak band located between 700 and 600 cm^−1^ is assigned to trioctahedral minerals. The strong band located at 530 cm^−1^ is assigned to Si–O-Al (octahedral Al) bending vibration modes (Madejova 2003). The microwave-assisted acid treatment causes modification in the bands of Kao-A spectrum, confirming that this acid treatment barely causes modifications in its structure as was observed in the XRD (Figs. 1 and 2) and SEM images (Fig. 4). The hydrothermal treatment under basic conditions causes a drastic modification of the ATR spectra since the typical bands of hydroxyl groups located between 3800 and 3500 cm^−1^ disappear. Regarding the 1300–500 cm^−1^ region, two bands are observed. The main one is shifted to a lower wavenumber value in comparison to the Kao and Kao-A samples, with a maximum at 970 cm^−1^ assigned to the Si–O stretching vibration mode; while the band with a maximum at *ca.* 550 cm^−1^ assigned to the Si–O-Al bending vibration mode (Wang et al. 2019).

**Fig. 6:**
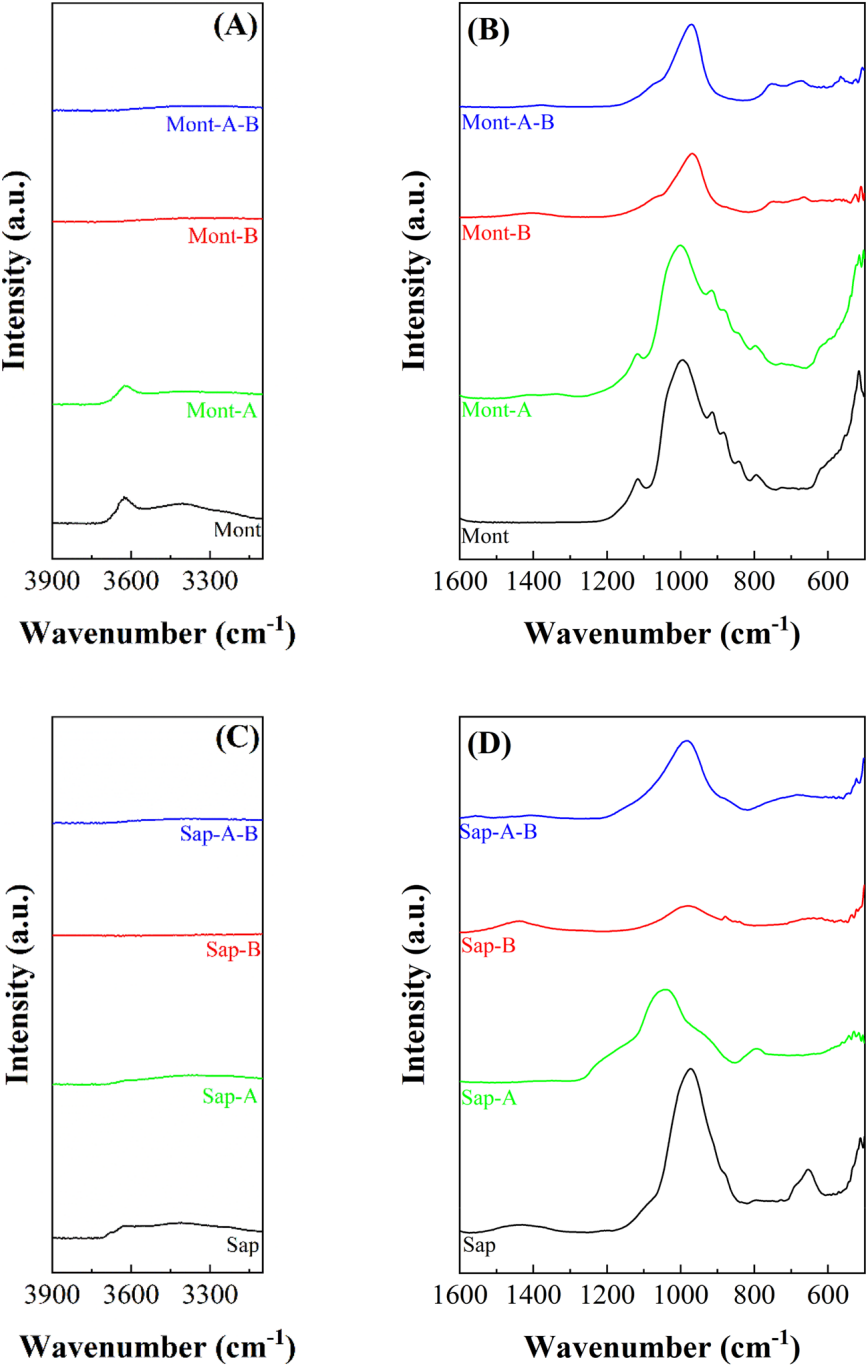
ATR spectra of montmorillonite (Mont), montmorillonite subjected to microwave-assisted acid treatment (Mont-A), montmorillonite subjected to hydrothermal treatment in basic conditions (Mont-B) and montmorillonite subjected to microwave-assisted acid treatment and then hydrothermal treatment in basic medium (Mont-A-B). ATR spectra of saponite (Sap), saponite subjected to microwave-assisted acid treatment (Sap-A), saponite subjected to hydrothermal treatment in basic conditions (Sap-B) and saponite subjected to microwave-assisted acid treatment and then hydrothermal treatment in basic medium (Sap-A-B)

The study of the Mont sample by ATR in the hydroxyl region (Fig. 6A) shows a band located around 3625 cm^−1^, which is assigned to Al(OH)Al-stretching vibrations (Cecilia et al. 2018a). Other authors have reported the presence of a band at higher wavenumber value attributed to the existence of pyrophyllite-like local structural fragments (Zviagina et al. 2004). The analysis of the 1300–400 cm^−1^ region (Fig. 6B) shows a main band located at *ca.* 1000 cm^−1^, which is assigned to the Si–O stretching mode. The bands located at 950–800 cm^−1^ are assigned to -OH bending bands of the dioctahedral clays (Al_2_OH or Fe_2_OH) (Madejova 2003) while the band located at 520 cm^−1^ is attributed to Si–O-Al bending vibrations (Wang et al. 2019). The analysis of the Mont-A spectrum confirms that the acid treatment does not affect the morphology of the clay since the ATR profile is maintained unaltered. After zeolitic treatment, the band related to hydroxyl stretching reactions disappears, while the number of bands located between 1300 and 500 cm^−1^ diminishes to the Si–O stretching band (965 cm^−1^). In addition, some impurities between 700 and 600 cm^−1^ appear, which may be ascribed to the Mg_2_OH bending vibration modes (Madejova 2003).

The ATR study of the saponite sample (Sap) (Fig. 6C) shows a band located at about 3675 cm^−1^, which can be ascribed to the -OH stretching vibration modes of the Mg(OH)_2_ groups located in the octahedral sheets of the smectite (Bisio et al. 2008), while the band located at about 3625 cm^−1^ can be assigned to -OH stretching modes of the Si(OH)Al groups due to the isomorphic substitutions of Si(IV) by Al(III) in its tetrahedral sheet (Cecilia et al. 2018a; Zviagina et al. 2004). Microwave-assisted acid treatment causes a loss of most of the bands attributed to the -OH stretching modes. In fact, only the presence of the band located at about 3625 cm^−1^, which is assigned to Si(OH)Al, is striking. Thus, from these data, it can be inferred that Mg-species of the octahedral sheet are leached while the small proportion of Al species located in the octahedral sheet seems to be more resistant to acid treatment. The analysis of the region between 1300 and 500 cm^−1^ (Fig. 6D) shows a main band whose maximum is located at 970 cm^−1^, which is attributed to the Si–O stretching mode, while the band located at 655 cm^−1^ is assigned to the Mg_3_OH bending vibration modes (Madejova 2003). Acid treatment provokes a shift in the Si–O stretching bands to higher wavenumber values due to the Si–O stretching mode of amorphous silica (Madejova 2003). This band is accompanied by another weak band located at about 800 cm^−1^, to which the presence of amorphous silica is also attributed (Madejova 2003). Regarding the samples treated under hydrothermal conditions in a basic medium, the typical band of the Si–O stretching mode assigned to the presence of an amorphous silica can be observed.

**Fig. 7:**
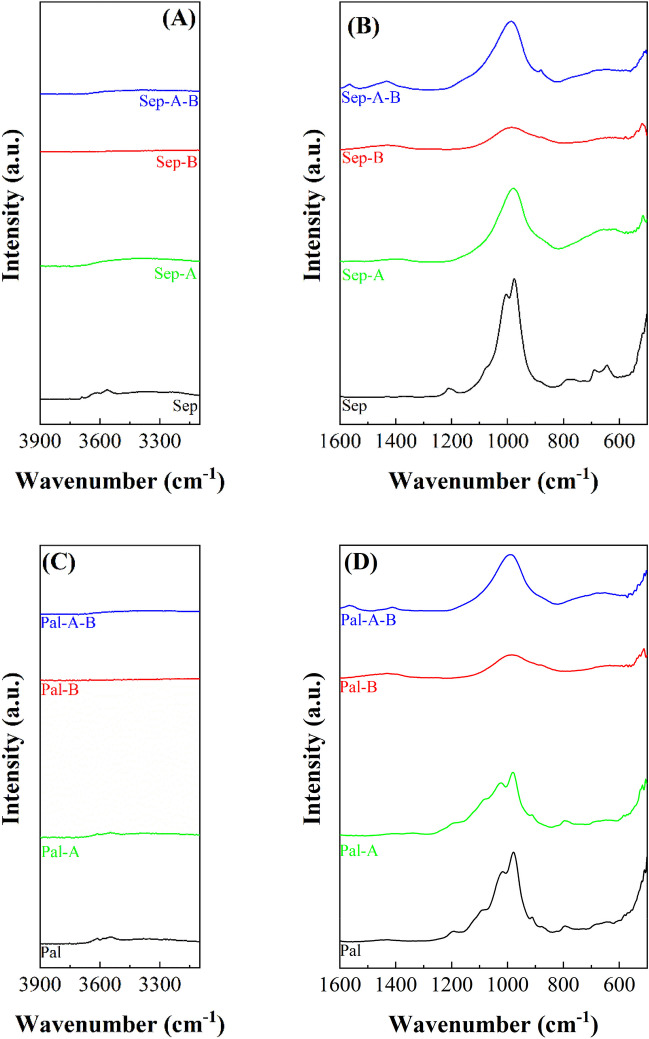
ATR spectra of sepiolite (Sep), sepiolite subjected to microwave-assisted acid treatment (Sep-A), sepiolite subjected to hydrothermal treatment in basic conditions (Sep-B) and sepiolite subjected to microwave-assisted acid treatment and then hydrothermal treatment in basic medium (Sep-A-B). ATR spectra of palygorskite (Pal), palygorskite subjected to microwave-assisted acid treatment (Pal-A), palygorskite subjected to hydrothermal treatment in basic conditions and palygorskite subjected to microwave-assisted acid treatment and then hydrothermal treatment in basic medium (Pal-A-B)

In the case of the raw sepiolite sample, the -OH stretching vibration (Fig. 7A) shows two peaks located at 3685 and 3625 cm^−1^, which are attributed to the stretching vibration modes of the -OH groups coordinated to Mg-species located in the octahedral sheet, while the band located at 3560 cm^−1^ is related to H_2_O coordinated with Mg-species (Franco et al. 2014). The region between 1600 and 500 cm^−1^ can be divided in two sections according to literature (Fig. 7B) (Frost et al. 2001): the bands between 1230 and 900 cm^−1^ are assigned to Si–O stretching vibration modes, where the maximum at 976 cm^−1^ corroborates the presence of Si–O-Mg in the octahedral sheet, while the bands between 700 and 600 cm^−1^ are attributed to M-OH translation (Frost et al. 2001). The study of the Sep samples after the microwave assisted acid treatment, Sep-A, reveals a modification in the profile between 1600 and 500 cm^−1^. It can be observed how the band of the Mg-OH band is deformed and the typical band of amorphous silica appears, as already observed in the case of the Sap-A sample. Similarly, the bands ascribed to Mg-OH deformation also disappear. When these samples are hydrothermally treated under basic conditions, the band is ascribed to the formation of amorphous silica. On the other hand, the absence of Al in this clay discards the presence of other bands ascribed Si–O-Al.

Finally, the study of raw Pal by ATR in the -OH stretching region (Fig. 7C) shows a band at about 3615 cm^−1^, which is typical of Al-species located in the octahedral sheet (Suárez and García-Romero 2006). Regarding the band located at about 3540 cm^−1^, previous authors have pointed out that this band is ascribed to Al–Fe-OH or Al–Mg-OH stretching vibration modes (Frost et al. 2001; Suárez and García-Romero 2006). In the region between 1600 and 500 cm^−1^ (Fig. 7D), three bands, with maxima located at 1187, 1118 and 1015 cm^−1^, are detected, which are attributed to periodically inverted Si–O-Si bonds in the tetrahedral sheet (Pardo-Canales et al. 2020; Wilson 2014). On the other hand, the band with a maximum about 980 cm^−1^ is assigned to the Si–O-Mg stretching vibration modes, as observed in the Sep sample. The band located about 910 and 880 cm^−1^ are assigned to Al–OH-Al and Al–OH-Fe (Suárez and García-Romero 2006). After the microwave-assisted acid treatment, Pal-A, it is observed how the bands are maintained, although those located in the -OH stretching region, between 3700 and 3500 cm^−1^, decrease in intensity. From these data, it can be inferred that the acid treatment only slightly affects the Pal structure, as suggested by XRD (Figs. 1 and 2) and SEM (Fig. 4). After basic treatment under hydrothermal conditions, most of the bands disappear, confirming that the structure is destroyed after acid treatment. Thus, a main band ascribed to the presence of amorphous silica can be observed. Likewise, the band with a maximum located at 660 cm^−1^ is attributed to Mg_2_OH bending vibration modes (Madejova 2003).

The analysis of the textural properties was carried out from CO_2_ adsorption–desorption isotherms (Table 2). As already mentioned, since the aim of this study is the use of clays and aluminosilicates obtained by hydrothermal treatment using basic conditions for the CO_2_ capture, CO_2_ has been used as probe molecule given its eased access to small pores, overcoming N_2_ thermodynamic limitations to assess microporosity. The study of the textural properties of raw clays shows how Kao, which displayed a higher order, exhibits the poorest pore volume and surface area. In the case of smectites (Mont and Sap), the surface area and pore volume are greater than those of Kao. Between them, the Sap sample displays a higher surface area and pore volume than the Mont sample due to its lower crystallinity or delamination, which promotes microporosity in its disordered structure. Regarding fibrous clays, the porosity of these materials must be located in their zeolitic channels, although the higher porosity of the Sep sample in comparison to that of Pal must be ascribed to the higher disorder of the Sep sample.

**Table 2:** Textural properties estimated from CO_2_ adsorption isotherms at 0 °C using the method Dubinin-Astakov of the raw phyllosilicates, phyllosilicates subjected to microwave-assisted acid treatment, phyllosilicates subjected to hydrothermal treatment in basic conditions and phyllosilicates subjected to microwave-assisted acid treatment and then hydrothermal treatment in basic conditions

Sample	Micropore capacity (mmol/g)	Micropore volumen (cm^3^/g)	Equivalent surface area (m^2^/g)
Kao	0.1393	0.0057	14
Kao-A	0.1190	0.0048	12
Kao-B	4.1826	0.1716	428
Kao-A-B	4.3900	0.1801	450
Mont	0.3687	0.0151	38
Mont-A	0.4269	0.0175	44
Mont-B	3.7138	0.1523	380
Mont-A-B	5.9548	0.2443	610
Sap	0.7795	0.0319	80
Sap-A	0.9832	0.0402	101
Sap-B	0.0953	0.0039	8
Sap-A-B	0.7440	0.0305	76
Sep	0.8150	0.0334	83
Sep-A	1.0121	0.0415	104
Sep-B	0.3347	0.0137	34
Sep-A-B	0.4553	0.0187	47
Pal	0.4732	0.0194	48
Pal-A	1.4916	0.0612	153
Pal-B	0.2660	0.0109	27
Pal-A-B	0.4826	0.0198	49

Samples obtained after the microwave-assisted acid treatment generally show a slight increase in microporosity, except for the Kao sample, which showed increased resistance to acid treatment as observed in the XRD data (Fig. 2). The improvement in microporosity is more pronounced in the case of the Pal sample. However, those samples subjected to a strong modification in their chemical composition (Sap-A and Sep-A) that eventually lead to a material rich in amorphous silica, hardly improves their microporosity. After the hydrothermal treatment in basic conditions of all samples, it can be observed how the textural properties only improve when Kao and Mont samples are used as starting materials, either in their raw or in their acid treatment-modified form, obtaining the best textural properties for the Mont sample treated through microwave-assisted acid treatment and then by hydrothermal treatment under basic conditions (Mont-A-B), which attained a micropore volume of 0.244 cm^3^/g and an equivalent surface area of 610 m^2^/g.

**Fig. 8:**
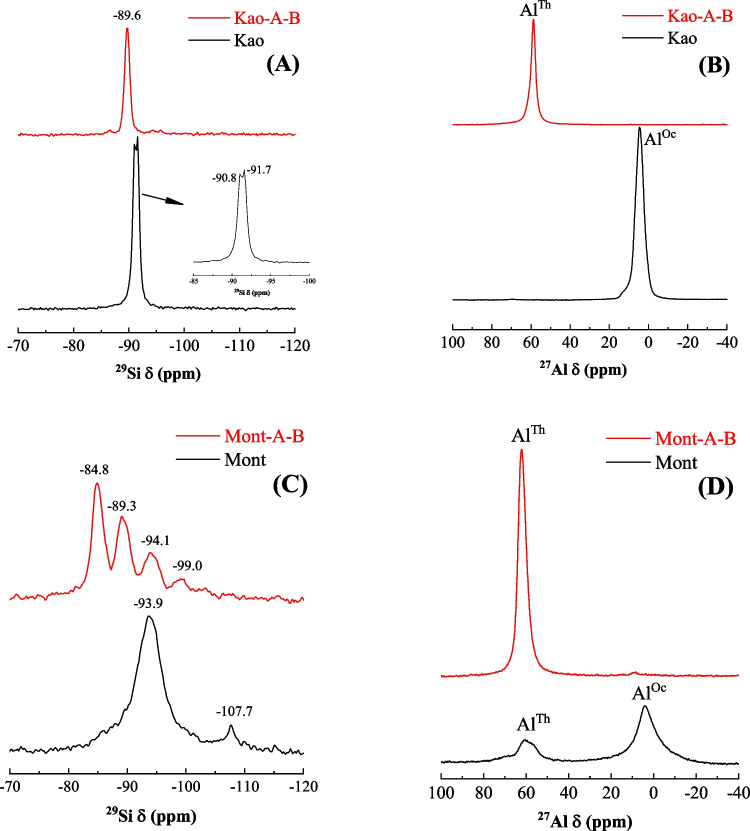
^29^Si NMR (**A**) and ^27^Al NMR (**B**) for the raw kaolinite and the kaolinite subjected to microwave-assisted acid treatment and then hydrothermal treatment in basic conditions. ^29^Si NMR (**C**) and ^27^Al NMR (**D**) for the raw montmorillonite and the montmorillonite subjected to microwave-assisted acid treatment and then hydrothermal treatment in basic conditions

Considering that the zeolites were only formed from the Kao and Mont samples, these samples were selected to be studied by ^27^Al and ^29^Si MAS ssNMR (Fig. 8). The analysis of the ^29^Si MAS ssNMR spectrum of the raw Kao (Fig. 8A) shows two signals located about − 90.8 and − 91.7 ppm. These signals are attributed to the silicate species located in the tetrahedral sheet. In the case of the raw Mont sample (Fig. 8C), the most intense signal located at − 93.9 ppm is also attributed to similar tetrahedral species interconnected with each other (Cecilia et al. 2022). Another weak band located at about − 107 ppm is also noteworthy, which could be ascribed to silicon bound in amorphous silica with a three-dimensional shape because of a small impurity of quartz (Breen et al. 1995). The analysis of the ^27^Al MAS ssNMR of the raw Kao sample (Fig. 8B) shows only one peak with a maximum at about 4 ppm typical of Al-species with octahedral coordination (Cecilia et al. 2018a; Hatakeyama et al. 2011). Interestingly, in the case of the Mont sample (Fig. 8D), this signal can be observed together with another one with a maximum at about 60 ppm, characteristic of Al-species with tetrahedral coordination (Hatakeyama et al. 2011). From these spectra, the presence of Al-pentacoordinated can be ruled out (Hatakeyama et al. 2011).

Hydrothermal treatment under acid and basic conditions causes a slight shift in the ^29^Si MAS NMR spectrum for the Kao-A-B sample to 89.7 ppm, attributed to Q^4^ sites (Si(4Al)) (Fig. 8A), in Zeolite A type materials (Shi et al. 1996). In this sample, contributions at higher and lower fields can also be elucidated, which indicates a change in the environment of the Si-species due to the incorporation of aluminum into the structure with a tetrahedral coordination (Kirkpatrick 1988). This fact is also corroborated by the ^27^Al signal, where only tetrahedral species are present (Fig. 8B).

In the case of the Mont-A-B sample (Fig. 8C), the ^29^Si MAS NMR spectrum is more complex since three main bands are observed at − 99.0, − 94.0, − 89.1 and − 84.8 ppm, which are assigned to Q^1^(Si(1Al)), Q^2^(Si(2Al)), Q^3^(Si(3Al)) and Q^4^(Si(4Al)) environments, respectively (Kirkpatrick 1988). In the case of the ^27^Al MAS NMR spectra, a band located at about 61 ppm is observed (Fig. 8D), confirming the presence of Al-species with tetrahedral coordination (Cecilia et al. 2018a).

## Adsorption studies

Once the clays and their respective materials synthesized from hydrothermal conditions in a basic medium had been characterized, the next step was the study of their CO_2_ adsorption capacity.

**Fig. 9:**
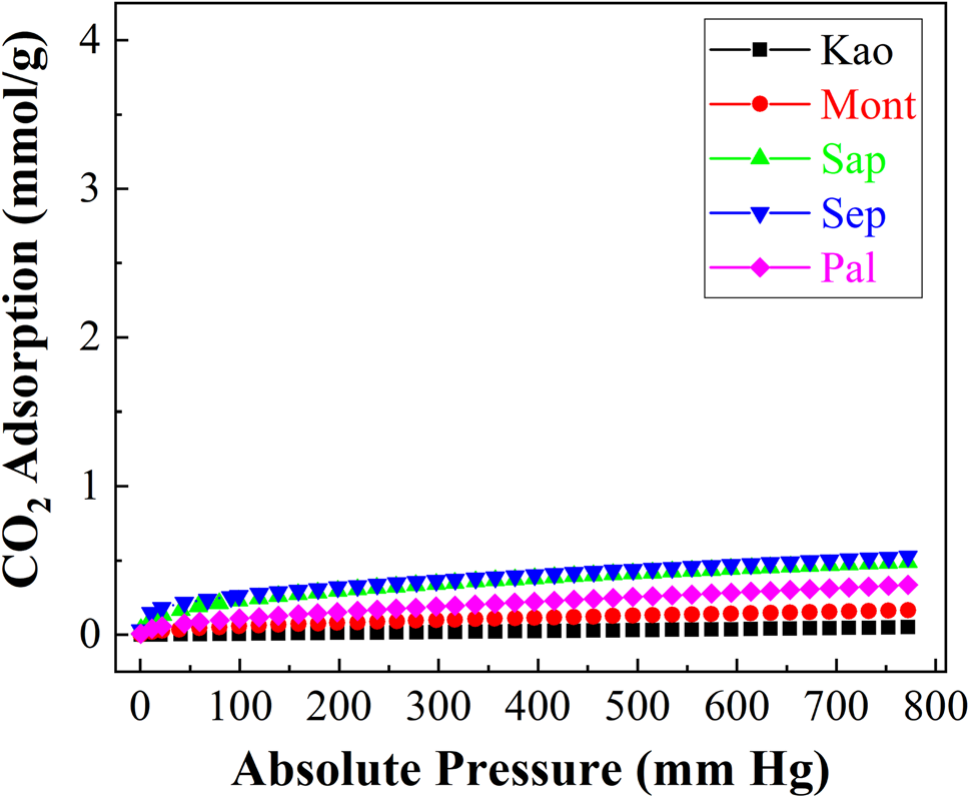
CO_2_ adsorption isotherms at 25 °C of the raw phyllosilicates

In the case of raw clay, the CO_2_-adsorption isotherms compiled in Fig. 9 show how the adsorption is quite linear, which suggests a weaker interaction than other adsorbents whose pore diameter is narrower. Also, it can be observed that samples with higher crystallinity and poorer textural properties, i.e. the Kao sample, display the lowest CO_2_ adsorption capacity, achieving a maximum value of 0.05 mmol/g at 25 °C and 760 mm of Hg. Between the smectites (Mont and Sap), the highest adsorption capacity is observed for the Sap sample. Considering that only the surface is responsible for the adsorption capacity of the materials since previous studies have discarded adsorption in the interlayer spacing (Chouikhi et al. 2021), the higher CO_2_-adsorption capacity takes place for the Sap sample, since this material shows less crystallinity in such a way that the CO_2_ molecules can be retained in the smaller pores of its disordered structure, which could resemble a house of cards structure. In fact, this low crystallinity of the Sap sample favors CO_2_ adsorption, a with an adsorption value of 0.49 mmol/g, while the Mont sample only reaches 0.16 mmol/g at 25 °C and 760 mm of Hg. As for the fibrous clays (Sep and Pal samples), a similar trend to that observed with smectites is observed, since the Sep sample, which displays less crystallinity, reaches a higher adsorption capacity. According to the morphology of fibrous clays where the tetrahedral sheet is periodically inverted, nanocavities are formed, thus it is expected that these microcavities could host a higher amount of CO_2_ molecules (Cecilia et al. 2018b; Suárez and García-Romero 2006). However, these fibrous materials show a low CO_2_ adsorption capacity to be competitive, since the Sep sample attains an adsorption capacity of 0.53 mmol/g while Pal, which showed a higher ordering by XRD, only achieves a CO_2_ adsorption capacity of 0.33 mmol/g at 25 °C and 760 mm of Hg. The isotherms were fitted to the Toth model as shown in Table 3. One of the most striking data obtained from the Toth equation is related to the b parameter, which defines the strength of the interaction between adsorbate and adsorbent, that is, the interaction between CO_2_ molecules and the clay. These data reveal that those materials with higher adsorption capacity and higher disorder in the clay structure also promote a stronger interaction CO_2_-clay, as shown by the b parameter. The t parameter defines the heterogeneity of the adsorbent. The data reported in Table 3 indicates that the adsorbents with more homogeneous adsorption sites are the fibrous clays probably due to the existence of nanocavities where CO_2_ adsorption must take place, as previously reported in literature (Cecilia et al. 2018b).

**Table 3:** Adsorption parameters, estimated from the Toth model, for the raw phyllosilicates

Sample	q_760_ (mmol/g)	q_m_ (mmol/g)	b (1/mmHg)	t	ARE (%)
Kao	0.05	1.31	4.73 x 10^-5^	0.27	3.68
Mont	0.16	1.98	4.88 x 10^-4^	0.34	2.55
Sap	0.49	3.57	2.74 x 10^-3^	0.14	2.73
Sep	0.53	3.80	7.59 x 10^-3^	0.45	3.89
Pal	0.33	2.23	5.62 x 10^-3^	0.38	5.36

**Fig. 10:**
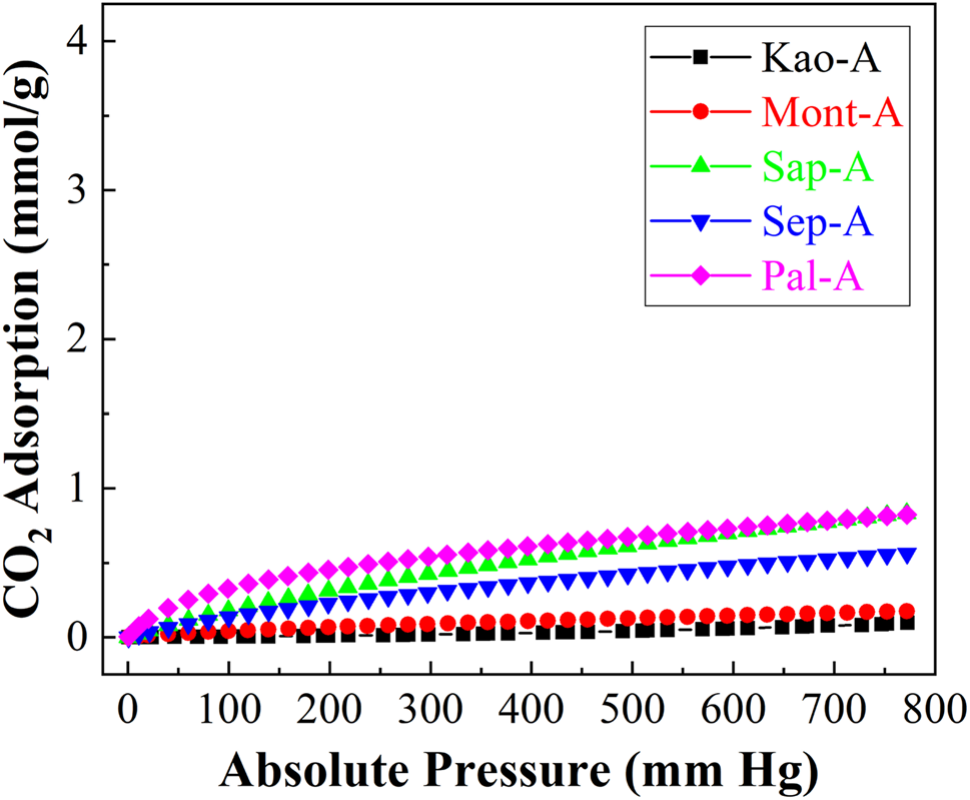
CO_2_ adsorption isotherms at 25 °C of the raw phyllosilicates subjected to microwave-assisted acid treatment

The CO_2_-adsorption capacity of clays after microwave-assisted acid treatment hardly improves their data (Fig. 10). Thus, both the Kao-A and the Mont-A samples maintain similar values to those observed before treatment (Fig. 9). These data agree with the characterization results, since these clays hardly suffer any modification after the acid treatment. In the case of the clays most prone to undergo modifications by acid treatment (Sap and Sep), it can be observed how the Sap-A sample significantly improves the CO_2_ adsorption capacity due to the formation of an amorphous material with higher porosity (Cecilia et al. 2018a), reaching a value of 0.82 mmol/g at 25 °C and 760 mm of Hg. In contrast, the Sep sample only slightly improves adsorption. In this sense, the collapse of the microchannels by the leaching of Mg-species must be the cause of this small improvement in the CO_2_ adsorption capacity. However, the Pal-A sample improves the CO_2_ adsorption capacity in comparison to the Pal sample (0.82 mmol/g and 0.49 mmol/g, respectively) probably because Pal is more resistant to acid treatment, therefore its fibrous structure does not collapse under this treatment. Regarding the fitting with the Toth model (Table 4), the b parameter is in the same range as that of raw clays, showing a poor affinity with CO_2_ molecules. The analysis of the t parameter is far from the unity, indicating that the adsorption process is not homogeneous but that there are preferential CO_2_ adsorption sites. In fact, phyllosilicates modified by microwave-assisted acid treatment display slightly higher values than those observed for raw clays probably due to partial leaching of the octahedral sheet after the acid treatment. This treatment is more effective in the case of saponite (Sap-A), which could be related to an increase in microporosity and the formation of a greater proportion of adsorption centers to capture CO_2_.

**Table 4:** Adsorption parameters, estimated from the Toth model, for the phyllosilicates subjected to microwave-assisted acid treatment

Sample	q_760_ (mmol/g)	q_m_ (mmol/g)	b (1/mmHg)	t	ARE (%)
Kao-A	0.09	1.91	2.18 x 10^-5^	0.32	2.87
Mont-A	0.18	2.11	2.23 x 10^-4^	0.39	2.58
Sap-A	0.82	3.97	6.22 x 10^-4^	0.32	0.47
Sep-A	0.57	2.78	3.25 x 10^-4^	0.48	0.81
Pal-A	0.82	4.07	6.06 x 10^-3^	0.42	1.80

**Fig. 11:**
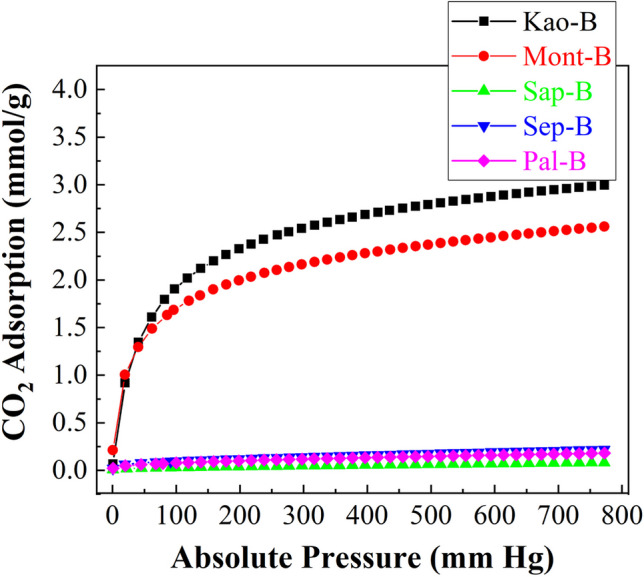
CO_2_ adsorption isotherms at 25 °C of the raw phyllosilicates subjected to hydrothermal in basic conditions

The study of the CO_2_ adsorption capacity of clay minerals after hydrothermal treatment under basic conditions (Fig. 11) causes a notable increase in the adsorption capacity of those materials synthesized from kaolinite and montmorillonite (samples Kao-B and Mont-B). These materials showed structures with high crystallinity, forming well-described zeolites such as 4A in the case of Kao-B and 13X for Mont-B. It is well known that both zeolites display narrow pores, which allows them to host a higher proportion of CO_2_ molecules, as observed by previous authors when synthesizing zeolites from other Si- and Al- sources (Barrer 1981). Thus, these samples reach a remarkably higher adsorption than that observed for their respective clays, achieving an adsorption capacity of 3.00 mmol/g for Kao-B while Mont-B shows an CO_2_-adsorption of 2.55 mmol/g at 25 °C and 760 mm of Hg.

In the case of Sap-B, Sep-B and Pal-B, the adsorption capacity is negligible in comparison to those of the Kao-B and Mont-B samples, so the formation of aluminosilicate with high crystallinity and narrow pore size is necessary to attain high CO_2_-adsorption capacity. For amorphous aluminosilicates, the polymerization of the aluminate and silicate species takes place in an uncontrolled manner, giving rise to aluminosilicate structures with undefined porosity and crystallinity, therefore with low microporosity and poor CO_2_-adsorption capacity. Moreover, the fitting of these isotherms to the Toth model (Table 5) shows that those aluminosilicates with defined crystallinity (Kao-B and Mont-B) have a higher affinity for the adsorbate, i.e. CO_2_ molecules, as indicated by their higher values of the parameter b. This trend is also observed for the t parameter since those adsorbents with higher microporosity and greater interaction with the adsorbate are also the one with the most homogeneous sites in which CO_2_ is preferentially adsorbent in the nanocages formed after the ordered assembly of the zeolites.

**Table 5:** Adsorption parameters, estimated from the Toth model, for the phyllosilicates subjected to hydrothermal treatment in basic conditions

Sample	q_760_ (mmol/g)	q_m_ (mmol/g)	b (1/mmHg)	t	ARE (%)
Kao-B	2.98	3.92	4.22 x 10^-1^	0.73	0.34
Mont-B	2.56	4.87	7.20 x 10^-1^	0.76	0.45
Sap-B	0.79	2.63	4.72 x 10^-3^	0.37	6.58
Sep-B	0.22	1.56	2.72 x 10^-2^	0.41	6.06
Pal-B	0.18	2.79	4.84 x 10^-2^	0.26	4.74

**Fig. 12:**
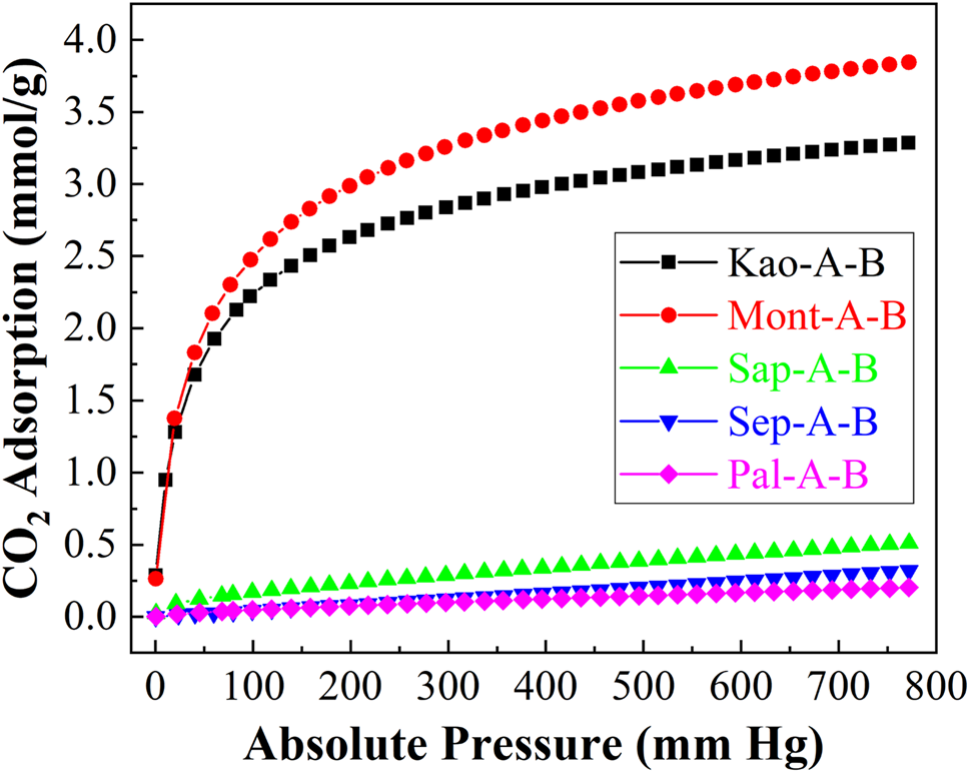
CO_2_ adsorption isotherms at 25 °C of the raw phyllosilicates subjected to microwave-assisted acid treatment and then hydrothermal in basic conditions

The analysis of the CO_2_ adsorption capacity for those samples subjected to an acid treatment and then a hydrothermal treatment under basic conditions (Fig. 12) reveals that it improves the CO_2_ capacity in the case of montmorillonite (Mont-A-B), obtaining a value of 3.87 mmol of CO_2_/g at 25 °C and 760 mm of Hg, while Kao-A-B only slightly improves the CO_2_-adsorption of the Kao-B sample. In this sense, the Mont sample showed a small proportion of impurities, which are removed after acid treatment. These impurities are insoluble in the basic media used for the synthesis of zeolites, so their presence has an adverse effect on their formation. After the acid treatment, these species that interfere with the synthesis of the aluminosilicates are removed, significantly improving the crystallinity of the obtained zeolite.

In the case of the kaolinite sample, it seems that the presence of impurities is lower, so the inclusion of an additional stage, such as the acid treatment, has a less decisive role in the zeolite synthesis than in the case of montmorillonite. For Sap-A-B, Sep-A-B and Pal-A-B, the CO_2_ adsorption values are much lower than in the other synthesized materials. In this sense, the removal of Mg-species by leaching through microwave-assisted acid treatment should avoid the presence of unwanted species in the synthesis of crystalline aluminosilicates. However, the absence or low contents of Al species prevent the formation of crystalline zeolites, at least under these synthetic conditions. This implies the formation of poorly ordered structures with low microporosity and therefore low CO_2_ capture capacity. The analysis of the adsorption isotherms according to the Toth model (Table 6) shows how those materials with higher adsorption capacity (Kao-A-B and Mont-A-B) also display a greater affinity for CO_2_ molecules. The analysis of the t parameter also indicates that the highly crystalline zeolites also display more homogeneous adsorption sites due to the formation of more ordered microporous adsorbents.

**Table 6:** Adsorption parameters, estimated from the Toth model, for the phyllosilicates subjected to microwave-assisted acid treatment and then hydrothermal treatment in basic conditions

Sample	q_760_ (mmol/g)	q_m_ (mmol/g)	b (1/mmHg)	t	ARE (%)
Kao-A-B	3.27	4.42	1.01x 10^-1^	0.84	1.58
Mont-A-B	3.85	7.36	4.73x 10^-1^	0.88	0.91
Sap-A-B	0.50	3.11	2.15x 10^-3^	0.23	6.01
Sep-A-B	0.32	2.39	6.78x 10^-5^	0.33	4.16
Pal-A-B	0.20	1.83	4.51 x 10^-4^	0.31	5.62

## Conclusions

Several phyllosilicates (Kao, Mont, Sap, Sep and Pal) have been selected as starting materials to try the synthesis of zeolites and their application in CO_2_ capture.

The analysis of the raw materials displays a poor adsorption capacity even in the case of the fibrous phyllosilicates where the presence of microcavities in their structure should promote higher adsorption capacity than Kao, Mont or Sap samples. Thus, the highest adsorption capacity for the starting materials was only of 0.53 mmol/g at 25 °C and a pressure of 760 mm of Hg for the Sep sample.

To improve the adsorption capacity, the phyllosilicates were subjected to a microwave-assisted acid treatment, where the trioctahedral clays, i.e. Sap and Sep, are more prone to suffer modification in its structure by the leaching of the Mg-species located in the octahedral sheet. Despite the improvement of the microporosity of the clays, the CO_2_ adsorption capacity is relatively low, reaching a maximum value of 0.82 mmol/g at 25 °C and 760 mm of Hg for Sap-A and Pal-A samples.

All these materials were subjected to a hydrothermal treatment in a basic medium to try the synthesis of zeolites with high microporosity. Among them, Kao, Mont and their respective materials subjected to acid treatment (Kao-A and Mont-A) were the only starting materials that gave rise to zeolites with high crystallinity. Specifically, the kaolinite-based materials gave rise to zeolite 4A while montmorillonite-based materials formed zeolite 13X. In both cases, the obtained zeolites are highly microporous, achieving a maximum CO_2_ adsorption capacity of 3.85 mmol/g at 25 °C and 760 mm of Hg for Mont-A-B sample. On the other hand, the aluminosilicates synthesized from Sap, Sep or Pal display poor CO_2_ adsorption capacity after the hydrothermal treatment under basic conditions. The low adsorption capacity is attributed to the high Si/Al molar ratio, which is not appropriate for a good assembly of the aluminate and silicate species to form crystalline zeolites with high microporosity.

## Data Availability

The authors confirm that all data generated or analyzed during this study are available from the corresponding author. These materials can be requested directly from the corresponding author if needed.
